# Cell-Specific Monitoring of Protein Synthesis *In Vivo*

**DOI:** 10.1371/journal.pone.0004547

**Published:** 2009-02-23

**Authors:** Nikos Kourtis, Nektarios Tavernarakis

**Affiliations:** Institute of Molecular Biology and Biotechnology, Foundation for Research and Technology-Hellas, Heraklion, Crete, Greece; National Institutes of Health, United States of America

## Abstract

Analysis of general and specific protein synthesis provides important information, relevant to cellular physiology and function. However, existing methodologies, involving metabolic labelling by incorporation of radioactive amino acids into nascent polypeptides, cannot be applied to monitor protein synthesis in specific cells or tissues, in live specimens. We have developed a novel approach for monitoring protein synthesis in specific cells or tissues, *in vivo*. Fluorescent reporter proteins such as GFP are expressed in specific cells and tissues of interest or throughout animals using appropriate promoters. Protein synthesis rates are assessed by following fluorescence recovery after partial photobleaching of the fluorophore at targeted sites. We evaluate the method by examining protein synthesis rates in diverse cell types of live, wild type or mRNA translation-defective *Caenorhabditis elegans* animals. Because it is non-invasive, our approach allows monitoring of protein synthesis in single cells or tissues with intrinsically different protein synthesis rates. Furthermore, it can be readily implemented in other organisms or cell culture systems.

## Introduction

Proper regulation of protein synthesis is critical for cell growth, cell proliferation and cell death. Protein synthesis involves a complex series of protein-protein and protein-RNA interactions which result in the formation of peptide bonds between amino acids, as encoded by the mRNA being translated. The rate of mRNA translation in eukaryotic cells is determined by a battery of mRNA translation factors [1]. Efficient proofreading and editing ensure the faithful decoding of mRNA into protein [2]. Deregulation of protein synthesis has been implicated in pathologies such as cancer and senescent decline [3], [4]. One of the most widely used approaches for measuring general protein synthesis rate is metabolic labelling, typically in the form of radioactive amino acid incorporation into nascent polypeptides [5], [6]. Overall protein synthesis activity can also be assessed by polysomal profiling, which provides a relative estimate of mRNA loading onto actively translating polyribosomes. In addition, polysomal profiling can be adapted to monitor translation of specific mRNAs [5]. These methodologies are useful for analyzing protein synthesis in cultured cells and in relatively homogenous, isolated tissues.

However, both metabolic labelling and polysomal profiling are hampered by several limitations. In particular, these approaches require relatively large amounts of biological material (cell or tissue mass) and do not allow monitoring of protein synthesis in specific sub-populations of cells or in single cells. Furthermore, they are associated with technical limitations that narrow their applicability. For example, efficient metabolic labelling in simple organisms, such as the nematode *Caenorhabditis elegans*, or in dissected mammalian tissues is technically challenging due to poor intake and uncontrolled or unequal distribution of the label throughout the animal or the tissue. Another source of variability comes from different intrinsic rates of protein synthesis in different tissues and cell types. Thus, significant changes in only specific cells or tissues that amount to a small fraction of the animal mass (such as the nervous system), may be obscured by more massive tissues (such as the intestine or the musculature). Polysomal profiling is hindered by similar issues. Finally, neither metabolic labelling nor polysomal profiling can be used to monitor protein synthesis in live animals.

We describe here a novel method for monitoring net protein synthesis rates in specific cells or tissues, based on fluorescence recovery after photobleaching (FRAP). This approach overcomes the drawbacks associated with biochemical, metabolic labelling methods and allows monitoring of protein synthesis in single cells or tissues, *in vivo*. Although in this study we implement the procedure for monitoring protein synthesis in *C. elegans*, the method can be readily adapted for applications in diverse organisms.

## Results

### Monitoring *de novo* protein synthesis by FRAP

Conventional FRAP applications usually involve highly localized photobleaching of fluorophores, within defined sub-cellular areas or compartments by means of a laser beam, under a confocal microscope [7], [8]. The objective is typically the assessment of lateral mobility or diffusion of proteins into the dark, photobleached area from surrounding regions [9], [10]. This analysis has the additional potential of providing indirect information about organelle continuity and protein trafficking. For the purpose of monitoring protein synthesis, we photobleached GFP-tagged, fluorescent proteins through the entire cell or tissue, to ensure that fluorescence recovery originates from *de novo* protein synthesis rather than from protein movement. We used animals expressing GFP throughout somatic tissues under the control of the *ife-2* gene promoter. *ife-2* encodes one of the five nematode eIF4E isoforms that functions in somatic tissues [4], [11]. eIF4E is a key mRNA translation initiation factor that binds the 7-methyl guanosine cap at the 5′ end of all nuclear mRNAs and determines the rate of cap-dependent protein synthesis [12]. Depending on the application, animals were either moving or anesthetized. Whole animals are then illuminated on an epifluorescence microscope, with a high power light source of the appropriate wavelength depending on the excitation spectrum of the fluorescent protein used.

**Figure 1:**
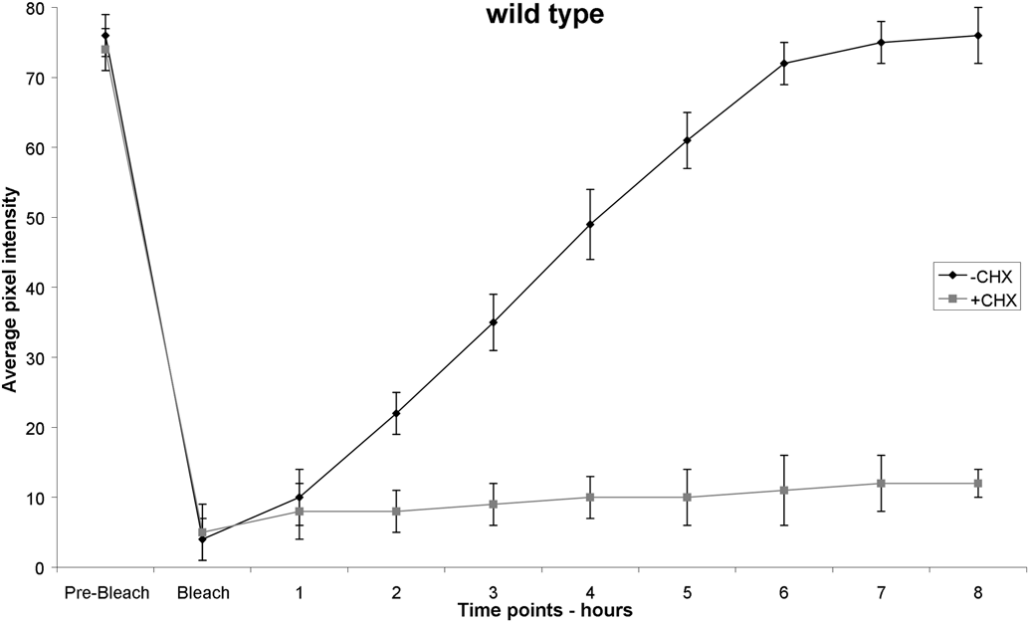
Transgenic animals expressing p*_ife-2_*GFP throughout somatic tissues, are subjected to a whole-animal photobleaching session for 8 min that reduces GFP fluorescence down to ∼10% of initial intensity (black line). Transgenic animals carry the *rol-6 (su1006)* allele as a co-transformation marker. Fluorescence is measured before photobleaching (Pre-Bleach) as well as immediately following the photobleaching session (Bleach). Subsequent recovery of fluorescence is followed by measurement of average pixel intensity at one-hour time intervals. Error bars represent SEM (4 independent experiments, 10 animals in each experiment). Treatment of animals with the specific protein synthesis blocker cycloheximide (CHX) at 500 µg/ml final concentration, diminishes fluorescence recovery (grey line).

By testing different extents of photobleaching, ranging from 5% to 50%, we find that accurate data are obtained by properly adjusting the duration and intensity of illumination, aiming to attain a level of photobleaching that reduces fluorophore emission down to between 10–20% of pre-bleaching intensity, without damaging specimens. We observed that depending on the cell or tissue of interest and the fluorescent marker used, a different optimal level of photobleaching may be required, which can be determined experimentally as the minimum photobleaching dose that will quench fluorescence of the reporter to a significant extend, without damaging the organism. Damage was assessed by observing behavioural traits (locomotion, egg laying, pharyngeal pumping, foraging) and reproductive capacity (fecundity and fertility), as a measure of irradiation toxicity. Control animals, where mRNA translation is blocked by treatment with the antibiotic cycloheximide, a potent and specific inhibitor of mRNA translation, were also included in the analysis. Following photobleaching, fluorescent cells or tissues of interest were photographed again and animals were allowed to recover on growth media. Recovery of fluorescence, which is indicative of new protein synthesis, was then monitored in targeted cells or tissues. To monitor fluorescence recovery, animals were photographed at specific time points using appropriate filter sets (see Materials and Methods). The images collected were used to calculate fluorescence intensity in cells and tissues under investigation, before and after photobleaching (Figure 1). Cycloheximide can also be used to discriminate between the contribution of new protein synthesis and protein diffusion in overall fluorescence recovery after photobleaching.

### Comparative analysis of protein synthesis rates between different genetic backgrounds

**Figure 2:**
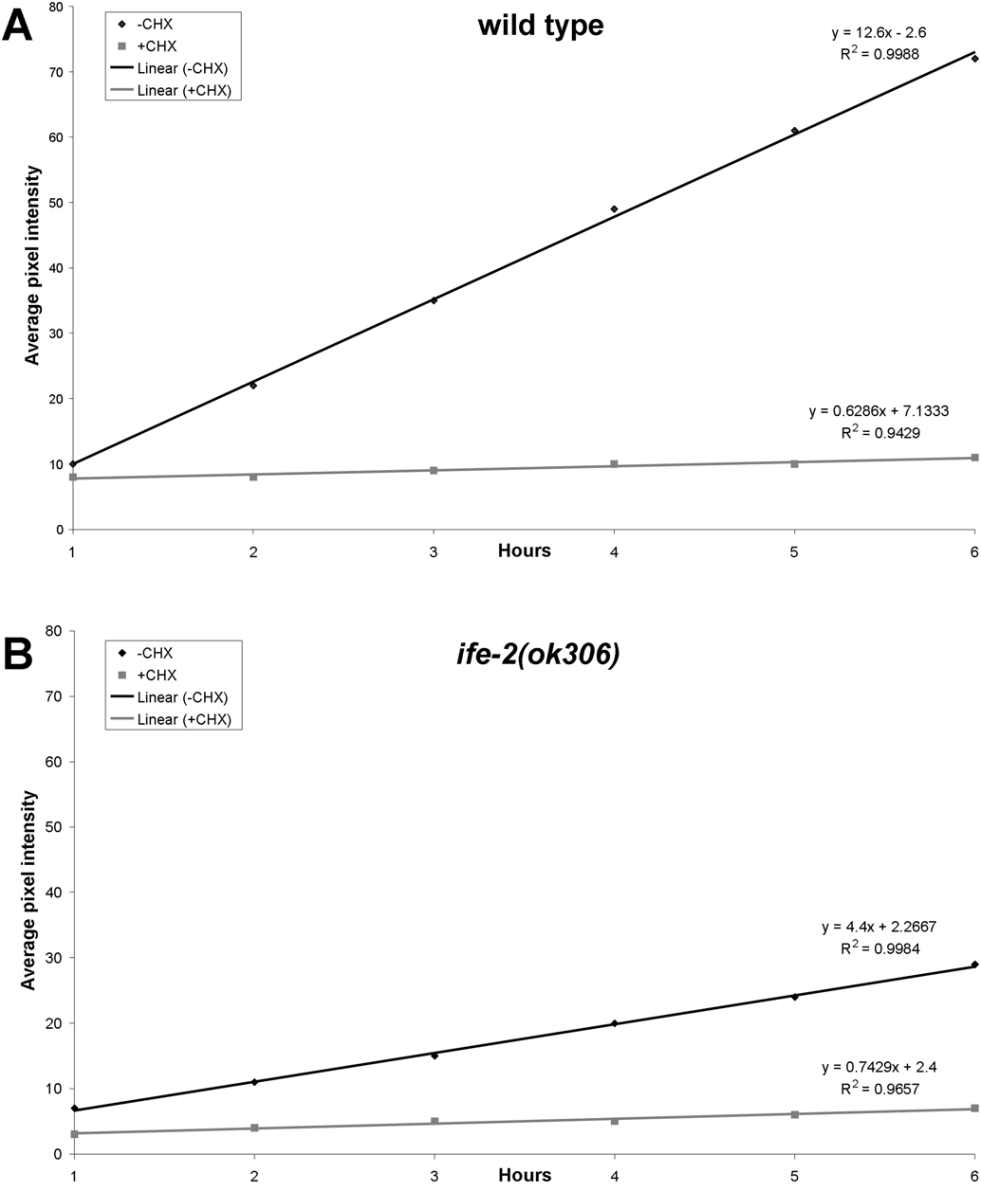
Regression analysis of fluorescence recovery in both wild type and IFE-2 deficient animals expressing p*_ife-2_*GFP throughout somatic tissues. Animals were photobleached and fluorescence recovery was followed as described in Materials and Methods. Best-fit lines are generated for average pixel intensity values obtained during the recovery phase for the indicated genetic backgrounds (A, wild type; B, *ife-2 (ok306)*; black lines). The respective equations describing best-fit lines as well as R^2^ values for each line are also shown. Line slope corresponds to the first derivative of fluorescent change within a time unit (Δf/dt), which is a measure of the recovery rate. Cycloheximide treatment (CHX) results in negligible recovery rate (grey lines).

**Figure 3:**
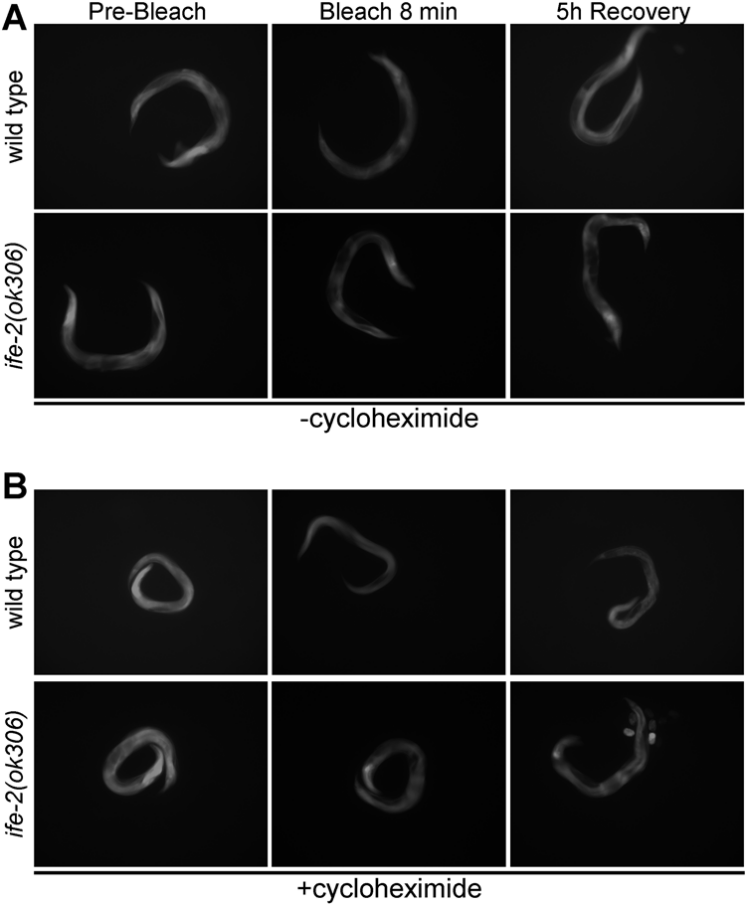
Representative images of roller, transgenic animals expressing p*_ife-2_*GFP throughout somatic tissues, before photobleaching, immediately following an 8 min whole-animal photobleaching session, and after a 5 h recovery period, in the absence (A) and presence (B) of cycloheximide at 500 µg/ml final concentration.

Assessment of protein synthesis rates is an important component of mRNA translation regulation studies. An array of protein factors facilitates the tight control of messenger RNA translation. In eukaryotes, the rate of cap dependent protein synthesis is mainly determined by the translation initiation factor eIF4E [12]. We compared protein synthesis rates between wild type animals and mutants deficient for the IFE-2, which is the main eIF4E isoform in *C. elegans* somatic tissues. After photobleaching, fluorescence recovery was monitored in transgenic animals expressing GFP throughout somatic tissues (Figure 2). The best-fit function that describes the recovery phase was generated by regression analysis. The slope of the best-fit lines provides quantification of the recovery rate in a manner similar to that of the conventional radioactive metabolic labelling experiments. Recovery is diminished in IFE-2-deficient animals or animals treated with cycloheximide (Figure 2A vs. Figure 2B), stressing the importance of IFE-2 factor in protein synthesis procedure. Representative images of animals analyzed are shown in Figure 3. We note that IFE-2 depletion does not affect transcription or the mRNA levels of the reporter fusions used [4], thus, allowing the comparative analysis of protein synthesis rates in wild type vs. animals that lack this factor. In addition, the protein synthesis inhibitor cycloheximide fully blocks fluorescence recovery after photobleaching. Thus, fluorescence recovery depends on *de novo* protein synthesis and is sensitive to genetic manipulations, which impact mRNA translation initiation. These observations suggest that the rate of fluorescence recovery is an indicator of the rate of protein synthesis.

### Cell and tissue-specific monitoring of protein synthesis

**Figure 4:**
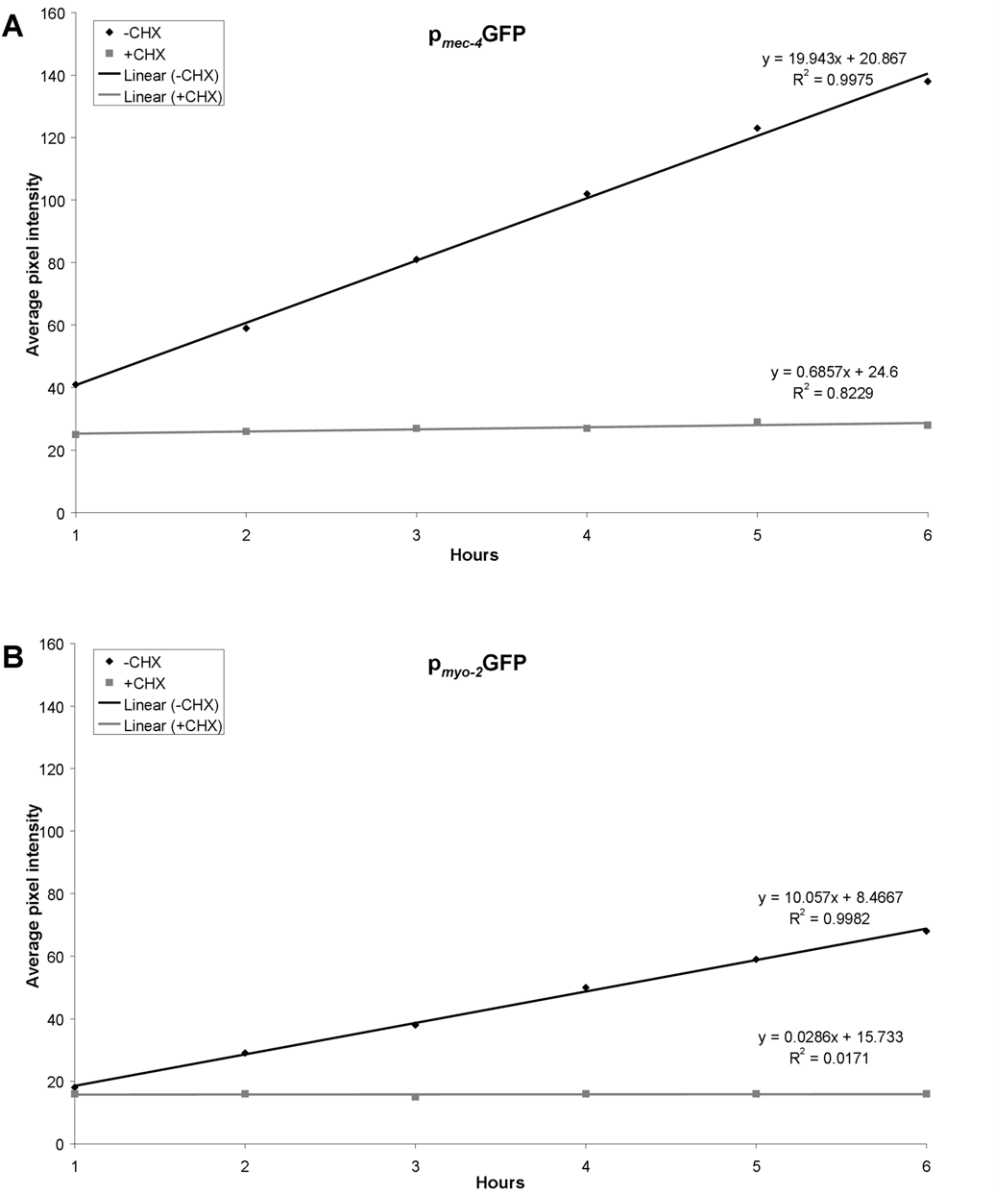
Regression analysis of fluorescence recovery in cell and tissue-specific level. (A) In wild type animals expressing p*_mec-4_*GFP in six specific neurons (the touch receptor neurons). Best-fit lines are generated for average pixel intensity values obtained during the recovery phase. Cycloheximide treatment (CHX) results in negligible recovery rate (compare black line: −CHX vs. grey line: +CHX). The respective equations describing best-fit lines as well as R^2^ values for each line are shown. Line slope corresponds to the first derivative of fluorescent change within a time unit (Δf/dt), which is a measure of the recovery rate. (B) In wild type animals expressing p*_myo-2_*GFP specifically in the pharyngeal muscles. Best-fit lines are generated for average pixel intensity values obtained during the recovery phase. Cycloheximide treatment (CHX) blocks recovery (compare black line: −CHX vs. grey line: +CHX). The respective equations describing best-fit lines as well as R^2^ values for each line are shown.

**Figure 5:**
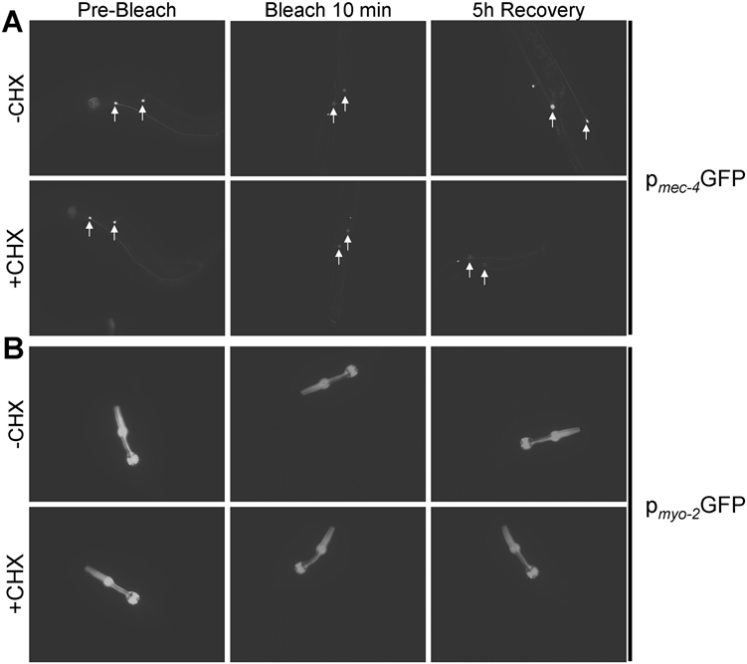
Representative images of transgenic animals before photobleaching, immediately following a 10 min whole-animal photobleaching session, and after a 5 h recovery period, in the absence (−CHX) and presence (+CHX) of cycloheximide. (A) Animals expressing p*_mec-4_*GFP specifically in the six touch receptor neurons. (B) Animals expressing p*_myo-2_*GFP specifically in pharyngeal muscle cells.

Different cell types and tissues show intrinsically different protein synthesis activities. Furthermore, fundamental biological processes such as development, differentiation and ageing influence mRNA translation in a cell and tissue specific manner [4], [13], [14]. Thus, the ability to determine protein synthesis rates in specific tissues or cells of interest, *in vivo*, within the context of the whole organisms is important for investigating the molecular mechanisms underlying differential mRNA translation regulation. We assessed the potential of the method for monitoring protein synthesis in different cell types, in live animals. To compare protein synthesis among different cell types, we followed fluorescence recovery after photobleaching, using two fluorescent reporter fusions expressed in specific cells and tissues. The first reporter is driven by the promoter of the *mec-4* gene, which is expressed specifically in the six touch receptor neurons [15]. The second reporter is expressed in pharyngeal muscles under the control of the *myo-2* promoter [16]. To avoid mRNA-specific effects on protein synthesis, both reporter fusions were designed to encode identical mRNAs, solely for GFP, with no other gene specific sequences. We find that fluorescence recovery after photobleaching is faster in neurons compared to muscle cells (Figure 4A vs. Figure 4B; for representative images of animals analyzed see Figure 5). In both cases inhibition of protein synthesis by cycloheximide blocks recovery (Figure 4). We observed a similar trend using different reporter fusions expressed in these two distinct sets of cells (not shown). These observations indicate that the rate of protein synthesis is higher in neurons compared to muscles (for additional paradigms, see Figure S2, S3, S4, S5, S6). Our findings are consistent with previous studies suggesting a lower protein synthesis activity in muscles [6], [14], [16], [17], [18].

## Discussion

We have developed a non-radioactive and non-invasive approach for monitoring protein synthesis *in vivo*, based on live imaging of fluorescence recovery after photobleaching. This method has the potential to allow monitoring of protein synthesis in single cells or tissues with intrinsically different protein synthesis rates (for example muscles vs. neurons or epithelial cells), *in vivo*. Such capacity is not possible with the currently available radioactive, metabolic labelling methods (Figure S1). These methods are exceedingly difficult to implement in *C. elegans* and in live animals in general (Materials and Methods S1). They involve hazardous manipulations of radioactive media (initial radioactive labelling of bacteria which are then used as food for worms grown on agar plates), and are confounded by several limitations, such as poor intake and uncontrolled or unequal distribution of the label throughout the tissue or animal. An additional important problem with both radioactive labelling and ribosomal profiling in whole organisms is that results are skewed by the intrinsically different rates of protein synthesis in different tissues and cell types. Ultimately, the contribution of massive tissues (muscles, intestine) obscures effects in small groups of cells such as neurons. This renders radioactive labelling data hard to interpret and ambiguous.

We have further evaluated our method in various contexts. Protein synthesis rate can be monitored for proteins in low abundance such as transcription factors (Figure S2), proteins that participate in highly organized cellular structures such as myofilaments (Figure S3), as well as for membrane ion channels (Figure S4). Both transcriptional and translational fusions to fluorophores are suitable for analysis. In addition, proteins localized in specific subcellular compartments are amenable to examination with the present protocol (Figure S5). Finally, we have tested the sensitivity of the method by analyzing the rates of protein synthesis of reporter fusions varying in length (Figure S6). In these experiments we found that recovery is temperature dependent and is slower at lower temperature. In addition, recovery is slower in aged animals versus young adults (data not shown). A relevant factor that also needs to be considered in interpreting the results is the half-life of GFP under the different temperatures and at different ages.

Our method provides a convenient and versatile alternative to burdensome and crude radioactive labelling procedures, and also allows new functionality and depth of analysis, at the single-cell level. This capacity now enables studies not feasible with conventional radioactive labelling methods, such as comparative analysis of protein synthesis between different cell types, *in vivo*. The role of specific mRNA translation factors in the regulation of protein synthesis in specific cells and tissues, under specific conditions or in specific developmental stages can be studied by assaying fluorescence recovery in mutant animals carrying genetic lesions in the corresponding genes or in wild type animals subjected to RNAi.

For the implementation of the method, we used two different means to interfere specifically with protein synthesis. First, mutations in i*fe-2*, the gene encoding a somatic isoform of the mRNA translation factor eIF4E [11] and second, cycloheximide, which is a potent and specific inhibitor of protein synthesis. Both these operations do not affect the mRNA levels of the reporters used. Although the experimental design we followed is based on GFP, other fluorescent markers such as DsRED or other variants could also be used. This flexibility circumvents potential autofluorescence background interference in the green channel due to the accumulation of lipofusin deposits (the age pigment) in the intestine of old animals. The use of red fluorescent reporters eliminates this problem. Because of its simplicity and flexibility, the method is likely to be applicable in a wide range of biological studies, in diverse experimental models and organisms. For example, localized changes in the rate protein synthesis occur during numerous biological phenomena such as learning and memory and in pathological situations such as Parkinson's and Alzheimer's diseases. The approach described here allows *in vivo* monitoring of such protein synthesis fluctuations, contributing to the investigation of such complex phenomena.

While, we have used the approach described here to obtain relative information on the rate of protein synthesis in animals with impaired mRNA translation, the method can be adapted to monitor other facets of gene expression such as DNA transcription, RNA splicing, mRNA transport and turnover, and protein maturation. For example, the effects of specific genetic or pharmacological manipulations, targeting these processes can be dissected in a similar manner.

## Materials and Methods

### Nematode strains

We followed standard procedures for *C. elegans* strain maintenance, crosses and other genetic manipulations [19], [20], [21], [22]. Nematode rearing temperature was kept at 20°C, unless noted otherwise. Some nematode strains were obtained by the *C. elegans* Knockout consortium [23] (Robert Barstead, Oklahoma Medical Research Foundation, USA) and the *Caenorhabditis* Genetics Center (Theresa Stiernagle, University of Minnesota, Minneapolis, USA). The following strains were used in this study: N2: wild type Bristol isolate, KX15: *ife-2 (ok306)X* [11], N2*Ex*[p*_ife-2_*IFE-2::GFP, pRF4] [4], N2*Ex*[p*_ife-2_*GFP, pRF4] [4], N2*Ex*[p*_myo-2_*GFP] [24], N2*Ex*[p*_mec-4_*GFP, pRF4] [25], N2*Ex*[p*_pqn-21_*GFP, pRF4] (this study), N2*Ex*[p*_gcy-5_*GFP, pRF4] [26], N2*Ex*[p*_sod-3_*GFP, pRF4] [27], N2*Ex*[p*_myo-3_*mitoGFP] [28], KX15*Ex*[p*_ife-2_*GFP, pRF4] [4], N2*Ex*[p*_myo-3_*MYO-3::GFP, pRF4] [29], N2*Ex*[p*_clp-1_*CLP-1::GFP, pRF4] [30], N2*Ex*[p*_asp-4_*ASP-4::GFP, pRF4] [30], N2*Ex*[p*_ife-2_*IFE-2::GFP, pRF4] [4], N2*Ex*[p*_pqn-21_*PQN-21::GFP, pRF4] (this study), N2*Ex*[p*_mec-4_*MEC-4::GFP, pRF4] [25], N2*Ex*[p*_mec-17_*LMP-1::GFP, pRF4] [31], N2*Ex*[p*_phb-1_*PHB-1::GFP, pRF4] [28]. Transgenic *C. elegans* strains expressing fluorescent proteins of choice, under the control of appropriate promoters that direct expression in specific cells or tissues of interest were created as described previously [32].

### Sample preparation, photobleaching and recovery

The FRAP procedure was performed either directly on a plate or on a coverslip. For worms expressing the fluorescent marker protein globally or in many tissues, we found it more convenient to perform the assay on a plate. In this case, single worms were transferred to fresh 35 mm plates, seeded with OP50 bacteria. A small bacterial spot in the centre of the plate made localization of the worm easier, while focusing the sample. By contrast, when we examined worms that expressed the fluorescent marker in individual cells, photographs of moving worms were hard to analyze. In this case, we spotted a drop of 15 µl M9 buffer on a microscope slide and placed the worm on the drop with the help of an eyelash glued on a pick. Then, we added a cover slip on the top of the drop. The weight of the cover slip was sufficient to keep the worm immobile during the procedure, without damaging it. To limit animal mobility, the mild anaesthetic levamisole that does not interfere with metabolic processes, was also used at final concentration of 1 mM [20], [21]. We avoided the commonly used sodium azide, which blocks the mitochondrial respiratory chain, perturbs energy production and is likely to interfere with the fluorescence recovery process, by hindering protein synthesis. An alternative strategy is to use suitable genetic mutants with limited mobility (uncoordinated, paralyzed). Care should be taken when designing the experiment to avoid genetic backgrounds that are likely to have an effect on protein synthesis. Generation and use of roller transgenic lines carrying the *rol-6 (su1006)* allele as co-transformation marker (plasmid RF4) helped confine animals in a small area of the plate during the photobleaching session.

Animals were photographed before photobleaching using a camera attached to the microscope (e.g. Axio Cam HR, Carl Zeiss). Images of fluorescent cells or tissues of interest were collected. Imaging parameters such as microscope and camera settings (lens and magnifier used, filters exposure time, resolution, etc.) were documented. All imaging parameters were kept identical throughout the experimental procedure. We performed photobleaching by using an epifluorescence, compound light microscope (e.g. Axioskop 2 Plus, Carl Zeiss, objective lenses: 10×, numerical aperture 0.3 and 20×, numerical aperture 0.5) equipped with a high power light source (HBO 100; 100 Watt mercury arc lamp; Osram, Munich, Germany) and the appropriate excitation/emission filter sets to photobleach the animal (488±10 nm band-pass excitation filter, 515±15 nm band-pass emission filter). For the applications described here 10 minutes of photobleaching reduced the initial emission intensity adequately (to within 10–30% of pre-bleach levels). The light intensity and the duration of the bleaching period were adjusted accordingly for the specific fluorophore, animal stage and cell or tissue under examination. The appropriate duration of irradiation required to reach an adequate extent of photobleaching, for different specimens was experimentally determined. At least 20 individual animals were processed for each experimental condition. The photobleaching period was kept identical for all animals tested. Proper photobleaching conditions (light intensity, duration) were set aiming to avoid injuring worms. The absolute level of fluorescence reduction by photobleaching is not important. We assessed damage to worms by looking for apparent changes in behaviour such as lethargy and movement defects or diminished responsiveness to touch, and for reduced fecundity in animals subjected to photobleaching. Animals showing signs of damage after photobleaching were excluded from further analysis.

Each animal was photographed immediately after photobleaching. Several images of cells or tissues of interest were collected. Animals were moved to fresh OP50-seeded NGM plates. To recover photobleached animals on a microscope slide, we added 100 µl of M9 at the edges of the cover slip and slide off the cover slip. These worms were also returned to an OP50-seeded NGM plate for recovery. Recovery timing started at this point. Fluorescence recovery was followed by photographing animals at defined time points. We used 1 hr intervals between successive photography sessions. A suitable time interval can be determined for each experimental application.

### Cycloheximide treatment

In order to verify that fluorescence recovery is due to new protein synthesis we used the antibiotic cycloheximide, a potent and specific inhibitor of protein synthesis. We added cycloheximide on top of OP50-seeded, 35 mm NGM plates to 500 µg/ml final concentration in the agar volume and allowed plates to dry. To kill bacteria on plates before adding cycloheximide we exposed bacterial lawns on NGM plates to UV radiation. We irradiated bacteria at 254 nm for 10 min at 100 mJ/cm^2^ in a UV crosslinker [13], [33]. Worms were transfered on cycloheximide-containing plates and incubated for 2 hours, at the growth temperature. After photobleaching, worms were returned in cycloheximide-containing plates during fluorescence recovery.

### Quantification of GFP emission and protein synthesis rate

To determine the average and maximum pixel intensity for each image of fluorescent cell or tissue of interest in the collected photomicrographs we processed images acquired in previous steps with the image processing software ImageJ (Rasband, W.S., ImageJ, U. S. National Institutes of Health, Bethesda, Maryland, USA, http://rsb.info.nih.gov/ij/, 1997–2006) [34]. For each cell, tissue or animal, images were converted to a pixel depth of 8 bit (256 shades of grey). To analyze the area of interest manually, we used the “freehand selection” tool to enclose the fluorescent area. Then, we selected the “measurement” command via the “analyze” drop-down menu to perform pixel intensity analysis. On occasion (area continuity, high contrast ratios), selection of the fluorescent area was done automatically. We selected “adjust” and then the “threshold” command, within the “image” drop-down menu of ImageJ. We adjusted the threshold until the region of interest was marked. Within the “analyze” drop-down menu, we selected the “analyze particles” command. By selecting “outlines” at the “show” drop-down menu, we checked whether measurements correspond to the area of interest. Average and maximum pixel intensity values were collected for each transgenic line and grouped into “Pre-bleach”, “Bleach” and “Recovery (n)”, where n is the time interval after photobleaching.

### Statistical analysis

Statistical analyses were carried out using the Prism software package (GraphPad Software Inc., San Diego, USA) and the Microsoft Office 2003 Excel software package (Microsoft Corporation, Redmond Washington USA). Mean values were compared using unpaired t tests. The R2 linear regression tool of the Microsoft Office 2003 Excel was used to generate best-fit lines corresponding to radioactive incorporation or fluorescence recovery rate. The Student's *t* test was used for two-way comparisons with a significance cut-off level of p<0.05. Analysis of variance (ANOVA) was used for comparisons of multiple groups of values, followed by Bonferroni-corrected multiple-group comparison posthoc *t* tests.

## Supporting Information

Figure S1 Monitoring of protein synthesis by conventional radioactive metabolic labeling. Incorporation of radioactive amino acids into nascent polypeptides in wild type animals at the indicated time points after growth on radioactive amino acid food source, either in the absence (black line) or in the presence (grey line) of the protein synthesis inhibitor cycloheximide. Best-fit lines are generated by regression analysis (the respective equations describing best-fit lines as well as R^2^ values for each line are indicated; cpm/µg: radioactive ^3^H disintegration counts per minute, per µg of protein after TCA precipitation).
(0.50 MB TIF)

Figure S2 Fluorescence recovery in wild type animals expressing p*_pqn-21_*PQN-21::GFP, a full-length transcription factor reporter fusion expressed at low levels (PQN-21; zinc-finger family; tight nuclear localization).
(0.48 MB TIF)

Figure S3 Regression analysis of fluorescence recovery in wild type animals expressing a full-length p*_myo-_*_3_MYO-3::GFP myosin fusion, which localizes in the myofilament lattice, specifically in the body wall muscles. Best-fit lines are generated for average pixel intensity values obtained during the recovery phase. The respective equations describing best-fit lines as well as R^2^ values for each line are shown.
(0.48 MB TIF)

Figure S4 Fluorescence recovery in wild type animals expressing a full-length p*_mec-4_*MEC-4::GFP ion channel fusion, which sorts through the Golgi and the endoplasmic reticulum and localizes on the plasma membrane, specifically in the six touch receptor neurons. Best-fit lines are generated for average pixel intensity values obtained during the recovery phase. The respective equations describing best-fit lines as well as R^2^ values for each line are shown.
(0.48 MB TIF)

Figure S5 Fluorescence recovery in wild type animals expressing either a p*_myo-3_*mitoGFP reporter fusion, localized in mitochondria of body wall muscles (grey line) or a p*_asp-4_*ASP-4::GFP reporter fusion, localized in lysosomes (black line). Best-fit lines are generated for average pixel intensity values obtained during the recovery phase. The respective equations describing best-fit lines as well as R^2^ values for each line are shown.
(0.52 MB TIF)

Figure S6 Regression analysis of fluorescence recovery in wild type animals expressing either a p*_sod-3_*GFP transcriptional reporter fusion (295 amino acids; black line), or a full-length, p*_ife-2_*IFE-2::GFP chimera (523 amino acids; dotted line), or a full-length p*_clp-1_*CLP-1::GFP fusion (1075 amino acids; grey line). Best-fit lines are generated for average pixel intensity values obtained during the recovery phase. The respective equations describing best-fit lines as well as R^2^ values for each line are shown.
(0.54 MB TIF)

Materials and Methods S1 (0.03 MB DOC)
