# Supplementary figures and images for: Cell-Specific Monitoring of Protein Synthesis In Vivo
Source: PLoS One. 2009 Feb 23;4(2):e4547. doi: 10.1371/journal.pone.0004547 (PMC2640430; doi:10.1371/journal.pone.0004547)

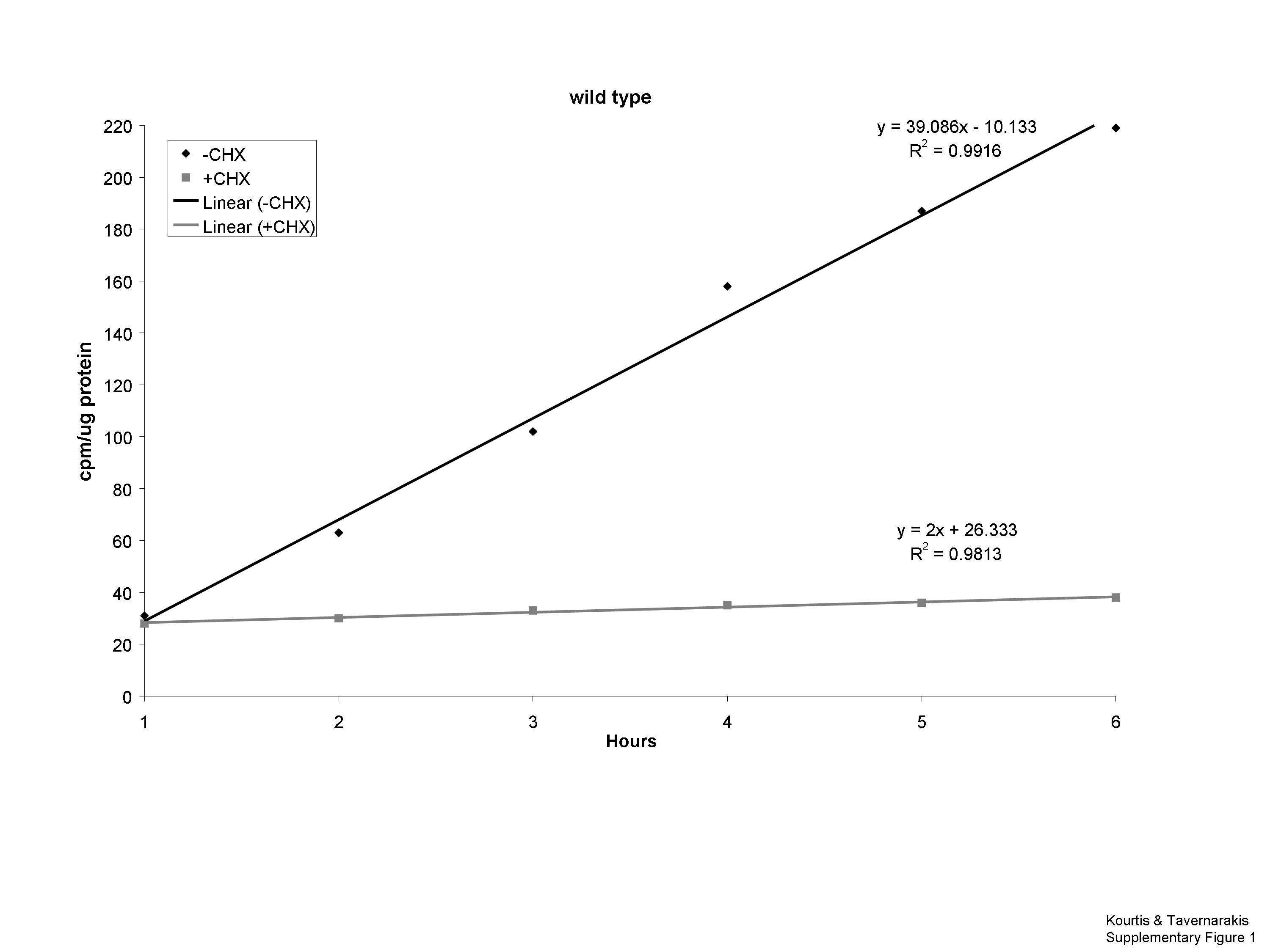

Supplement: Figure S1 — Monitoring of protein synthesis by conventional radioactive metabolic labeling. Incorporation of radioactive amino acids into nascent polypeptides in wild type animals at the indicated time points after growth on radioactive amino acid food source, either in the absence (black line) or in the presence (grey line) of the protein synthesis inhibitor cycloheximide. Best-fit lines are generated by regression analysis (the respective equations describing best-fit lines as well as R2 values for each line are indicated; cpm/µg: radioactive 3H disintegration counts per minute, per µg of protein after TCA precipitation). (0.50 MB TIF) [file pone.0004547.s001.tif]

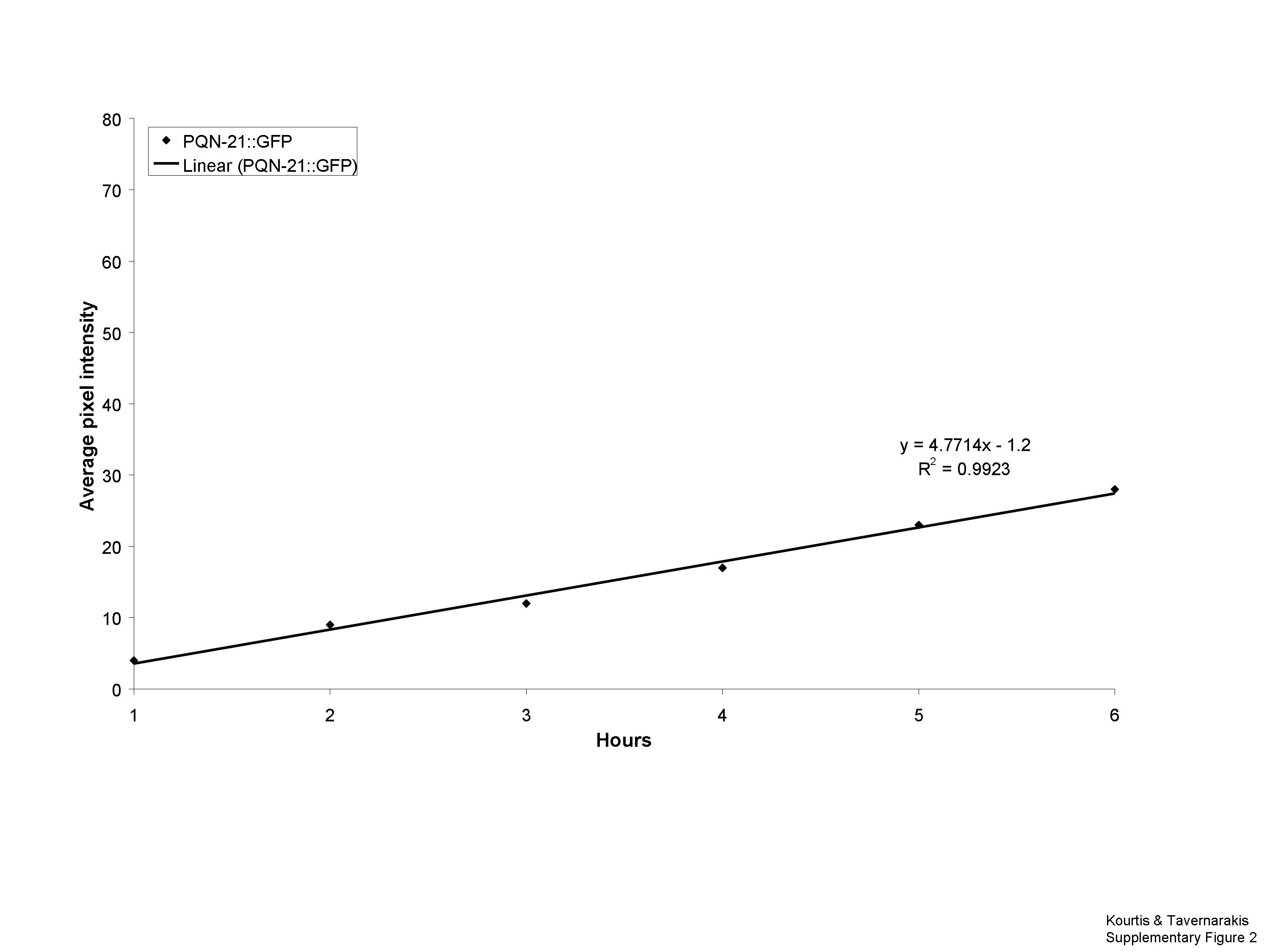

Supplement: Figure S2 — Fluorescence recovery in wild type animals expressing ppqn-21PQN-21::GFP, a full-length transcription factor reporter fusion expressed at low levels (PQN-21; zinc-finger family; tight nuclear localization). (0.48 MB TIF) [file pone.0004547.s002.tif]

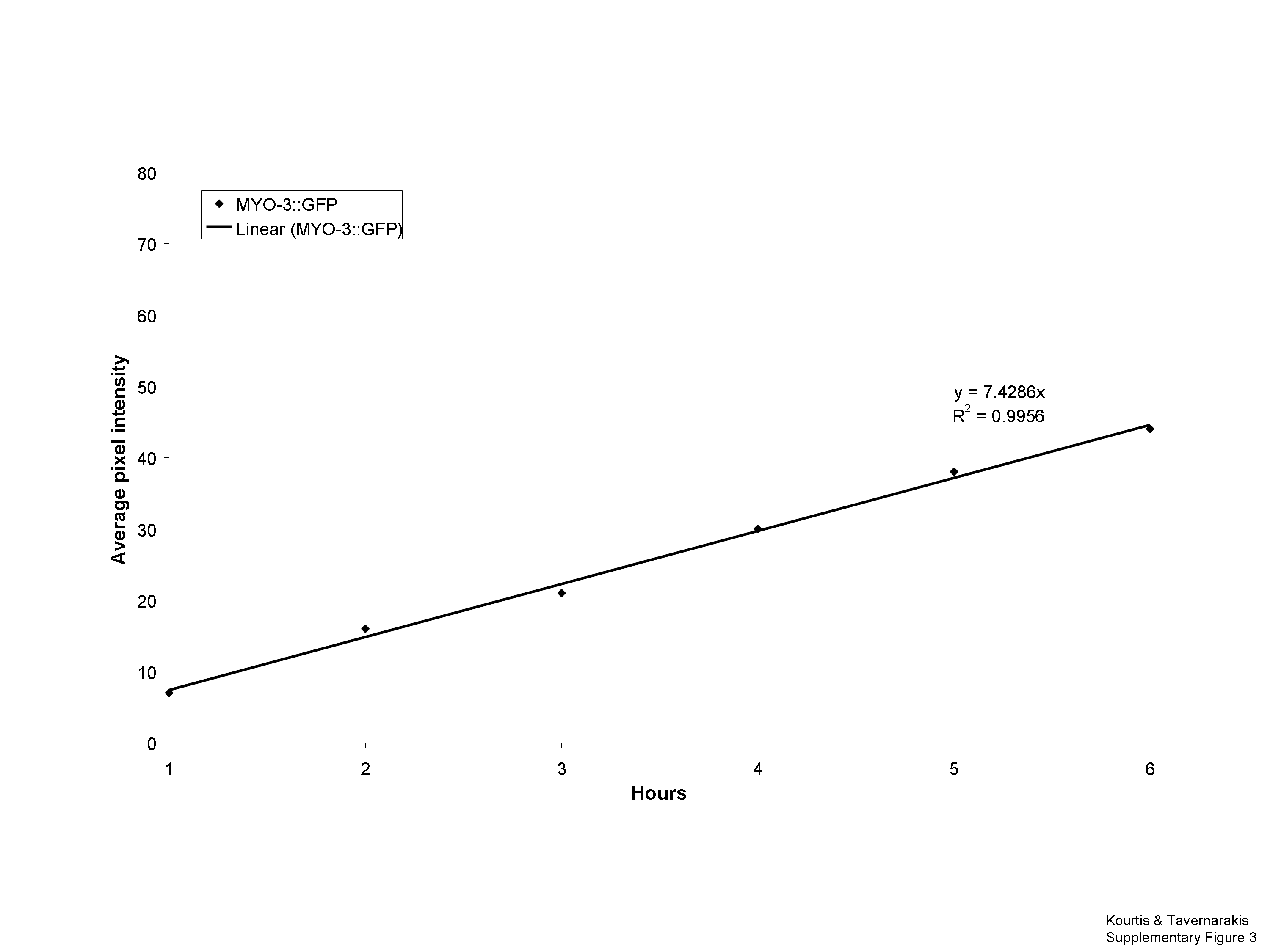

Supplement: Figure S3 — Regression analysis of fluorescence recovery in wild type animals expressing a full-length pmyo- 3MYO-3::GFP myosin fusion, which localizes in the myofilament lattice, specifically in the body wall muscles. Best-fit lines are generated for average pixel intensity values obtained during the recovery phase. The respective equations describing best-fit lines as well as R2 values for each line are shown. (0.48 MB TIF) [file pone.0004547.s003.tif]

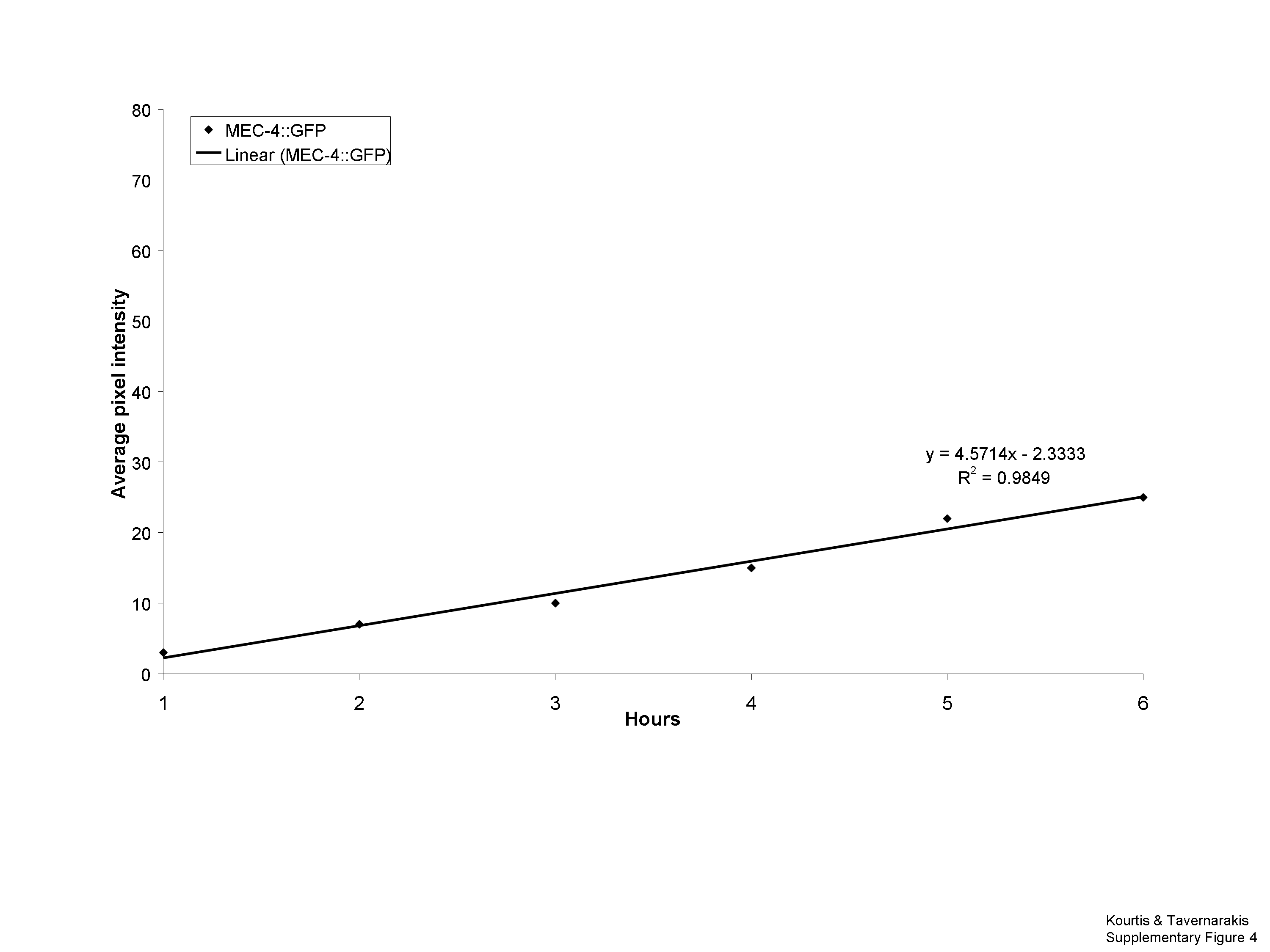

Supplement: Figure S4 — Fluorescence recovery in wild type animals expressing a full-length pmec-4MEC-4::GFP ion channel fusion, which sorts through the Golgi and the endoplasmic reticulum and localizes on the plasma membrane, specifically in the six touch receptor neurons. Best-fit lines are generated for average pixel intensity values obtained during the recovery phase. The respective equations describing best-fit lines as well as R2 values for each line are shown. (0.48 MB TIF) [file pone.0004547.s004.tif]

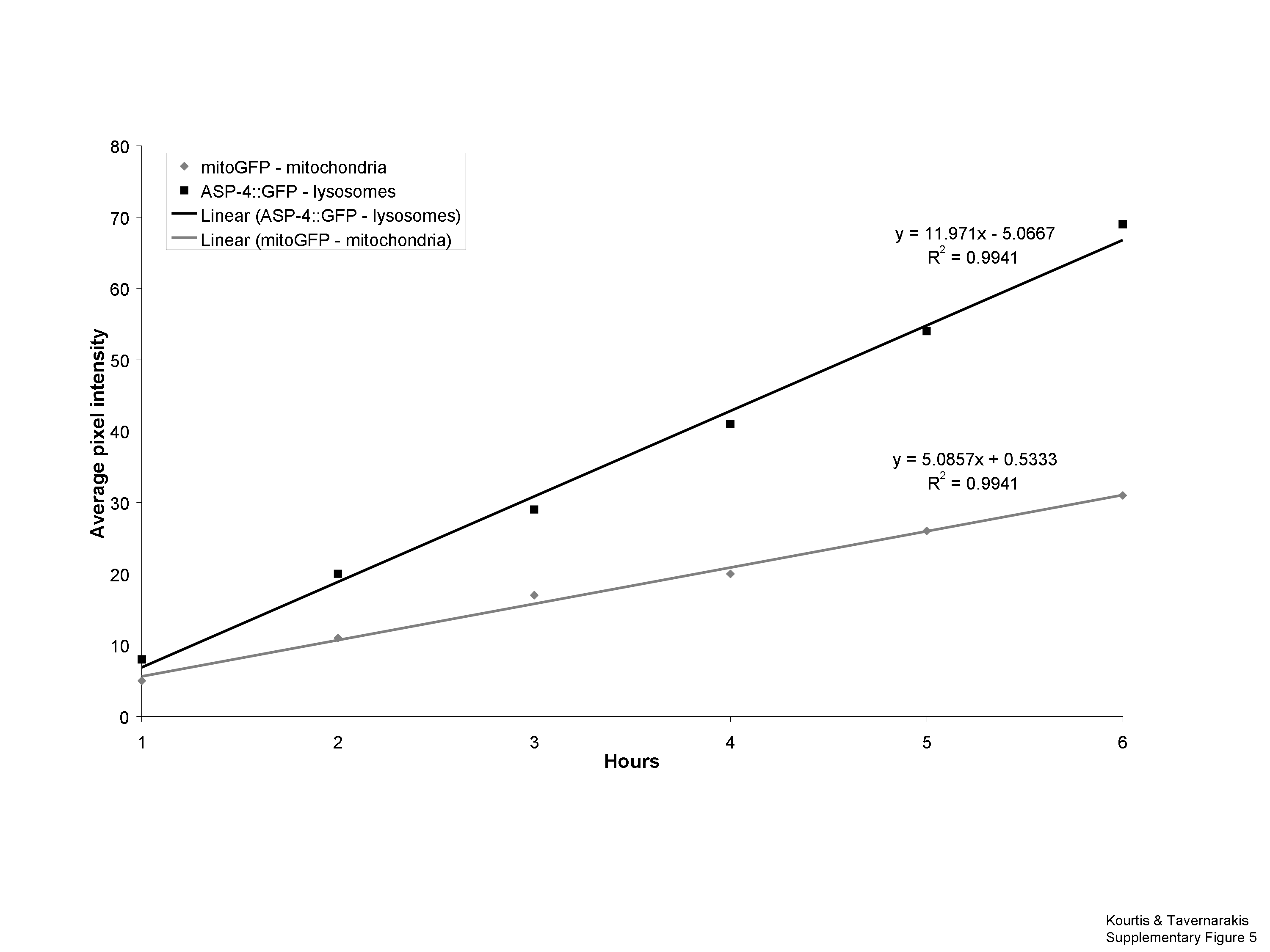

Supplement: Figure S5 — Fluorescence recovery in wild type animals expressing either a pmyo-3mitoGFP reporter fusion, localized in mitochondria of body wall muscles (grey line) or a pasp-4ASP-4::GFP reporter fusion, localized in lysosomes (black line). Best-fit lines are generated for average pixel intensity values obtained during the recovery phase. The respective equations describing best-fit lines as well as R2 values for each line are shown. (0.52 MB TIF) [file pone.0004547.s005.tif]

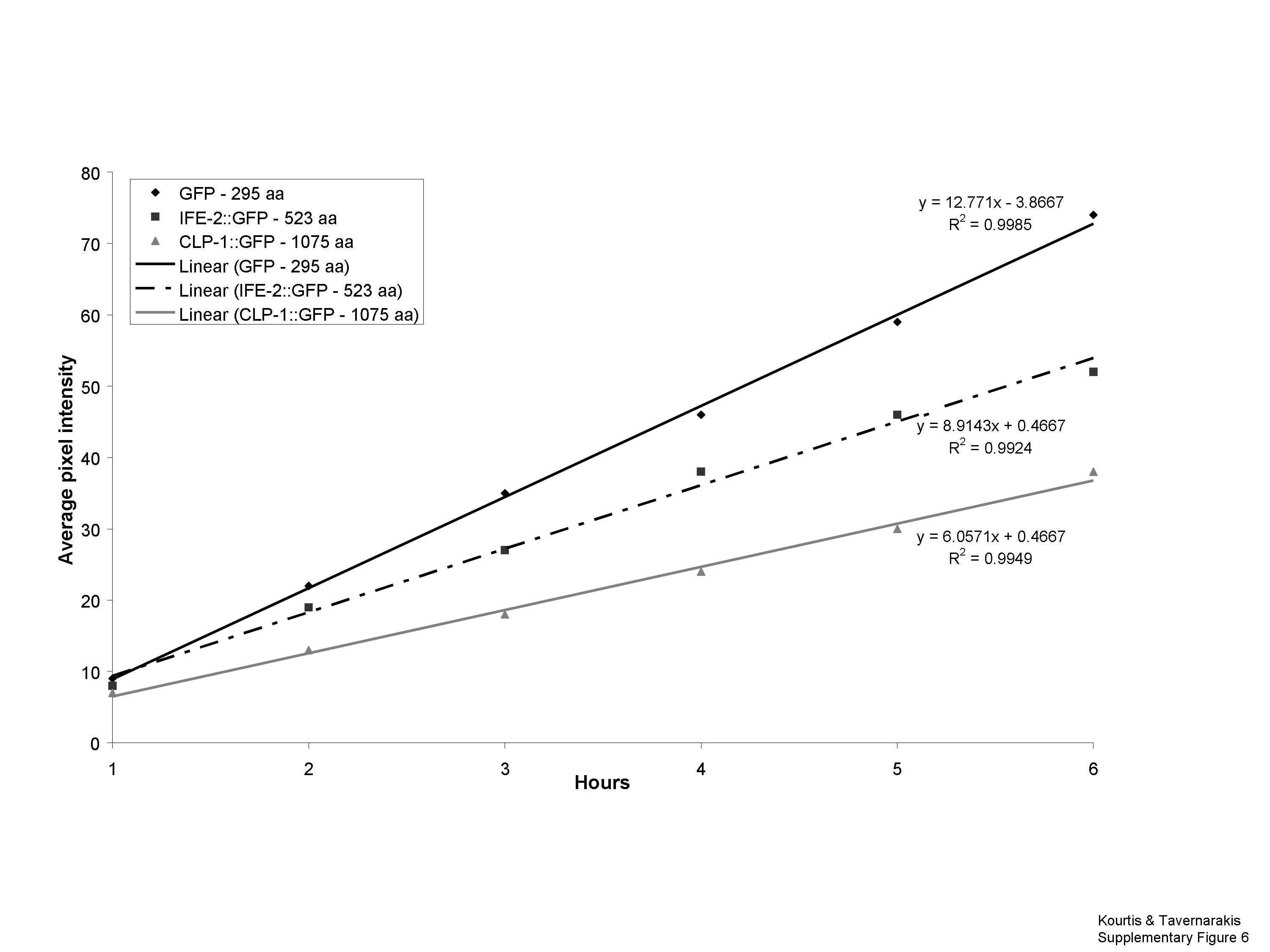

Supplement: Figure S6 — Regression analysis of fluorescence recovery in wild type animals expressing either a psod-3GFP transcriptional reporter fusion (295 amino acids; black line), or a full-length, pife-2IFE-2::GFP chimera (523 amino acids; dotted line), or a full-length pclp-1CLP-1::GFP fusion (1075 amino acids; grey line). Best-fit lines are generated for average pixel intensity values obtained during the recovery phase. The respective equations describing best-fit lines as well as R2 values for each line are shown. (0.54 MB TIF) [file pone.0004547.s006.tif]
