# Supplementary material for: Cell-Specific Monitoring of Protein Synthesis In Vivo
Source: PLoS One. 2009 Feb 23;4(2):e4547. doi: 10.1371/journal.pone.0004547 (PMC2640430; doi:10.1371/journal.pone.0004547)
Supplement: Materials and Methods S1 — (0.03 MB DOC) [file pone.0004547.s007.doc]

**Cell-specific monitoring of protein synthesis *in vivo***

**Nikos Kourtis and Nektarios Tavernarakis**

**Supporting Information**

**Materials and Methods S1**

**Radioactive metabolic labeling protein synthesis assays.** To assay protein synthesis by radioactive metabolic labeling, synchronized nematode populations, at the young–adult stage, were grown on UV-killed OP50 bacteria at 25^o^C and labelled with ^3^H-leucine (100μCi/plate, L-[3,4,5-^3^H (N)]-Leucine, PerkinElmer, Wellesley, USA). Control animal populations were grown in the presence of the protein synthesis inhibitor cycloheximide (500μg/ml) for 3.5 hours prior to labelling. For each time point, animals were harvested, washed three times in M9 buffer containing 100μg/ml cycloheximide and lysed in 25mM Tris-HCl (pH=7.4), 150mM NaCl and 0.25% SDS, at 100^o^C for 10 min. Insoluble cell debris were removed by centrifugation at 13,000rpm for 5min. Unincorporated ^3^H-leucine was removed by TCA precipitation. Total protein concentrations were determined by the BioRad Protein Assay (BioRad, Hercules, USA), whereas the amount of ^3^H-leucine incorporated per μg of protein was determined by liquid scintillation counting following TCA precipitation.
